# From Vegetable Waste to By-Product: Rheological Analysis of a Potential High-Protein Vegetable Burger

**DOI:** 10.3390/gels11121017

**Published:** 2025-12-18

**Authors:** Olga Mileti, Francesco Filice, Francesca R. Lupi, Domenico Gabriele, Noemi Baldino

**Affiliations:** Department of Information, Modeling, Electronics and System Engineering (D.I.M.E.S.), University of Calabria, Via P. Bucci, Cubo 39C, I-87036 Rende, CS, Italy; o.mileti@dimes.unical.it (O.M.); francesco.filice@dimes.unical.it (F.F.); f.lupi@unical.it (F.R.L.); domenico.gabriele@unical.it (D.G.)

**Keywords:** proteins, plant protein, meat alternative

## Abstract

(1) Foods with attractive shapes have been receiving increasing interest from researchers, particularly for foods for children. The ability to particularize foods by imparting attractive aspects to nutritious and less attractive food ingredients, such as vegetables or proteins, is an interesting challenge for the food industry. In this context, the rheological characteristics of food doughs are fundamental for obtaining form-forming foods that are able to maintain a shape of their own. (2) Broccoli, pumpkin, carrot and zucchini wastes (stems, leaves, and off-gauge veggies), which are still rich in nutrients, from the food industry were used in this work to enrich burgers with vegetable proteins. The doughs were characterized by rheological analysis using a frequency sweep test and a temperature ramp test. They were also shaped with attractive molds and baked. (3) From the frequency sweep test, the formulation with brown rice proteins resulted in better consistency; all samples showed a solid-like behavior. (4) Workable doughs were formulated using vegetal wastes from the food industry. Among the proteins used, those from brown rice were found to be the most suitable for the preparation of a vegetable burger.

## 1. Introduction

Meat consumption has been important to humans for centuries, both for nutrition and evolution. Meats are a source of protein and fats that are essential for growth [1].

Population growth has led to unsustainable meat consumption, both in terms of land use, animal exploitation, and CO_2_ emissions associated with this entire supply chain and a loss of natural biodiversity [2]. Vegetarian diets are more environmentally sustainable than meat-based diets [2]. For these reasons, the demand for alternative protein sources has increased, leading to the development of plant-based alternatives that satisfy consumers who, for ethical reasons, do not consume meat products [3]. Consuming plant-based products also has positive effects on health, maintaining good cholesterol control, limiting type 2 diabetes pathology, and limiting cardiovascular diseases [1,2,4]. From a nutritional standpoint, nearly all plant foods contain all nine essential amino acids [5]. The difference is in the proportions compared to meat, dairy products, and eggs. The key difference is that certain plant foods have one or more essential amino acids in lower proportions than what is required by the body (known as limiting amino acids). Animal proteins typically have proportions that more closely match human needs [5]. Nevertheless, the excess presence of some essential amino acids exceeding FAO recommendations creates a compensatory effect that ensures the appropriate protein consumption [6,7]. Specifically, the synergistic intake of varied protein sources facilitates the assimilation of the essential amino acids necessary for the body to function [8]. Moreover, soy stands out among all vegetable-based proteins for its superior essential amino acid profile [9]. These plant proteins also have an abundance of non-essential amino acids, which are integral to the maturation of the body’s metabolic functions [9].

Successful market substitution requires the products to meet both nutritional requirements and consumer textural preferences. Consequently, a comprehensive understanding of the rheological characteristics of doughs used for meat alternatives, such as vegetable burgers, is essential for replicating the sensory attributes of traditional hamburgers. Animal-based meat is an anisotropic material composed of interconnected muscle fibers, connective tissue, fat, and water; this complex internal structure is difficult to replicate in plant alternatives. This is a problem for non-fibrous pea- and soy-based meat alternatives [10]. Texture Profile Analysis (TPA) is widely used in studies to evaluate the sensory characteristics—such as stiffness, hardness, cohesiveness, springiness, resilience, and chewiness—of these products, facilitating the creation of similar substitutes [10,11,12]. This method is capable of accurately representing the properties of food products but lacks standardization.

Some alternative foods have been developed using textured vegetable proteins as structuring agents, and the desired consistency is achieved by using hydrocolloids such as methylcellulose [1,13,14]. In a 2019 study, the use of microbial transglutaminase (MTGase) was proposed to cross-link soy proteins in vegetable burger formulations, thereby improving the sensory texture of the samples [15]. Specifically, the vegetable burgers were prepared using onions, starch, gluten, carrageenan, and xanthan for structure, along with soy protein and textured soy protein. The addition of MTGase forms bonds within the glutamine fraction of the soy protein, resulting in the formation of high-molecular-weight polymers.

Texture analysis confirmed that incorporating MTGase into the formulations enhanced the cohesiveness of the batters and significantly improved the hardness, springiness, and chewiness of the final product. Furthermore, MTGase enhanced the gel-forming ability of the soy protein, leading to an increased network density within the soy protein-based product [15].

Analyses of Western consumer behavior confirm that the successful market substitution of conventional meat with plant-based alternatives depends on two crucial requirements: the product must convincingly imitate the textural and sensorial qualities of meat and accurately align with established meal expectations [13]. Unfortunately, some alternative product formulations have resulted in sensory characteristics that poorly mimic those of meat products, exhibiting lower values for hardness, chewiness, and gumminess compared to their meat-based counterparts [1].

For this reason, “hybrid” products—which are partial meat substitutes—are often developed. These products use a combination of meat and plant-based ingredients to reduce overall meat consumption while simultaneously improving the nutritional value [16].

In light of the above, research on plant-based products is necessary to improve their acceptance and consumption. From a circular economy perspective, utilizing waste from the vegetable processing industry—which is still rich in nutrients—presents an excellent opportunity. Specifically, vegetable trimmings are an attractive plant-based source that currently lack a defined role in production; their reuse is therefore crucial for both resource optimization and advancing the circular economy.

Controlling the rheological properties of the vegetable burger mixture before shaping and cooking is crucial for creating stable and predictable systems. Therefore, this research analyzed the rheological behavior of formulations made with various vegetable proteins.

For the study, vegetable burger doughs were prepared using vegetable cuttings sourced from nearby companies. Vegetable proteins were utilized to structure the preparations, while citrus fiber was employed to achieve a suitable consistency.

Soy, pea, and hemp proteins were used in this study to enrich the burger. In particular, soy, and hemp proteins were used because they are complete proteins, meaning that they contain all nine essential amino acids, while pea protein was used because of its functional properties.

The prepared mixtures underwent comprehensive rheological characterization to evaluate their consistency and structure. Finally, the resulting doughs were shaped and cooked to assess their final appearance.

## 2. Results and Discussion

The initial analysis was performed on a classic hamburger sample to study its consistency and structure in rheological terms: complex modulus $G^{*}$ and phase angle. The vegetable samples were then studied and compared with the classic meat-sed product. The study was carried out by analyzing the moduli in terms of frequency and temperature variation to evaluate the consistency, the structure, and stability of the products. The data obtained were used to gather information about strength and structural parameters, which are useful for comparing the preparations and evaluating the mechanical characteristics of the mixtures prepared.

### 2.1. Hamburger Characterization

**Figure 1:**
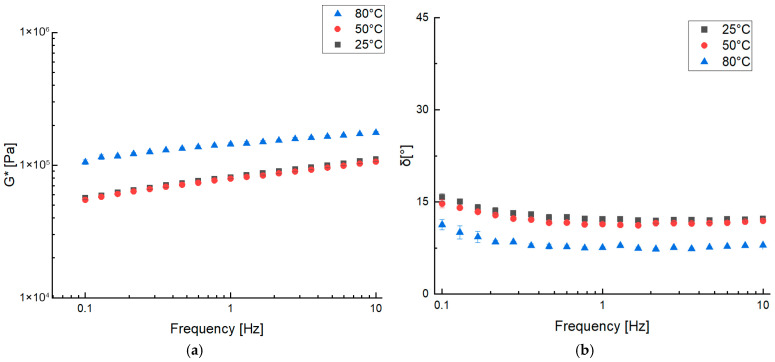
Frequency sweep test of Ham at 25 °C, 50 °C, and 80 °C measuring complex dynamic modulus $G^{*}$ (**a**) and phase angle δ (**b**).

A hamburger sample (Ham) was used as the standard, which was prepared in the laboratory according to a commercial recipe for hamburger and compare it to the veggie burgers. Figure 1 shows the results obtained from frequency sweep tests conducted at 25 °C, 50 °C, and 80 °C in terms of $G^{*}$ (a) and δ (b). The frequency sweep test graphs reveal a slight increase in the complex modulus ($G^{*}$) with rising frequency across all conditions. The material exhibited similar consistency at 25 °C and 50 °C, as indicated by the comparable $G^{*}$ values. In contrast, a significant increase in the complex modulus was noted at 80 °C, which is presumably due to the structuring of meat proteins. This phenomenon aligns with the denaturation range of myosin and actin, the primary myofibrillar protein components, which occurs between 40 °C and 80 °C [17]. Furthermore, the phase angle behavior remained similar at 25 °C and 50 °C, but a pronounced decrease was evident at 80 °C, consistent with a more solid-like behavior and structure of the sample.

**Figure 2:**
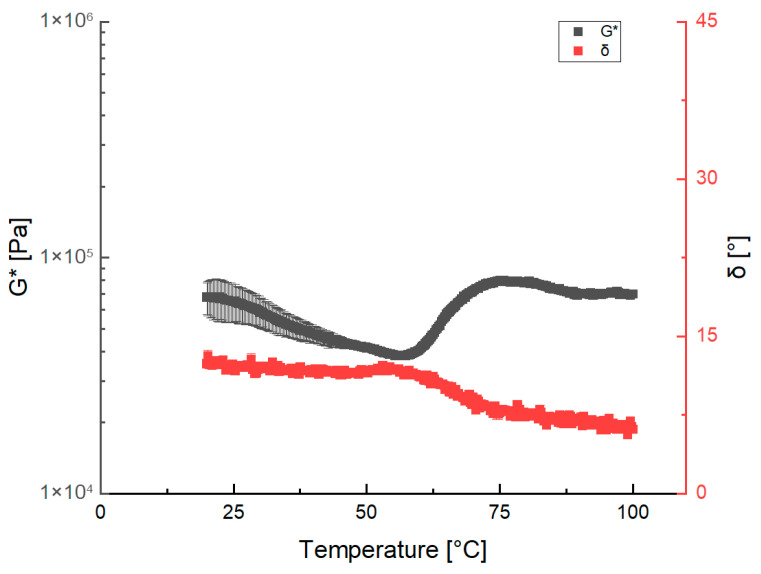
Ham temperature ramp test in terms of the complex dynamic modulus, $G^{*}$, and the phase angle, δ.

Additionally, a time cure test was employed to perform the temperature variation analysis, with the results presented in Figure 2.

The temperature analysis (Figure 2) demonstrates a gradual reduction in the complex modulus with increasing temperature, suggesting a reduction in consistency. This attenuation is likely due to kinetic phenomena that promote softening of the internal bonds and the material’s structural framework. This softening and weakening effect occurs across many types of dough, including gluten-based systems, specifically in the temperature range preceding starch gelatinization [18]. Nevertheless, the $G^{*}$ modulus displayed an abrupt increase between 50 °C and 75 °C, which signals a major rearrangement of the internal structure. This phenomenon is consistent with the denaturation and structuring of proteins that occur within this temperature range, as previously discussed in [17]. Similar to the behavior of $G^{*}$, the phase angle remained relatively constant across the 25 °C to 55 °C range, yet it exhibited a notable peak centered around 55 °C. Considering the increase in $G^{*}$ and the peak observed in the δ angle, it is evident that protein denaturation led to structuring of the sample.

The trend of $G^{*}$ as a function of temperature is consistent with the data obtained from the frequency sweeps at the three studied temperatures. Further increases in temperature up to 100 °C did not induce significant changes in $G^{*}$, which stabilized between 70 and 80 kPa. The phase angle inspection revealed no significant variation within the 25 °C to 55 °C range. In the temperature range above 55 °C, there was a peak near 88 °C and a decrease in the δ value was observed, which again suggests sample structuration. Finally, the very small decrease in δ between 88 °C and 100 °C strongly indicates the formation of a stable, highly structured network. The analysis carried out on the classic hamburger sample is a reference point for the subsequent plant-based material processing and preparation.

### 2.2. Veggie Burger Characterization

**Figure 3:**
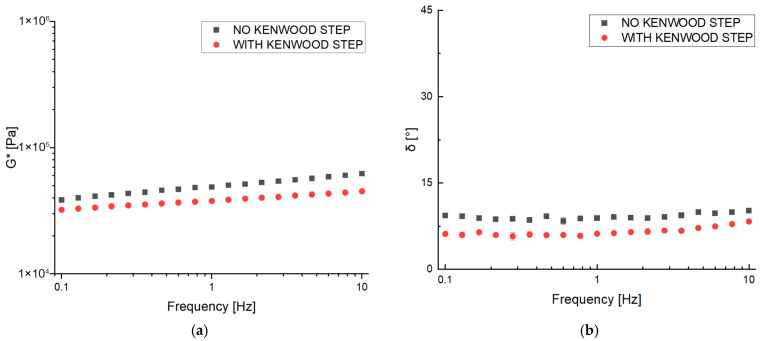
Frequency sweep test of ZBR at 25 °C measuring complex dynamic modulus $G^{*}$ (**a**) and phase angle δ (**b**), with and without Kenwood mixing step.

The ZBR sample was studied both as is and after being blended with a Kenwood mixer to observe the structural changes; the results of the process ($G^{*}$ and phase angle) are reported in Figure 3.

The addition of the homogenization step using the Kenwood mixer (Kenwood Cooking Chef XL KCL95.424SI, Kenwood Limited, New Lane, Havant, UK) resulted in less consistent and more homogeneous mixtures and structured material. Specifically, lower values of $G^{*}$ and δ were obtained after homogenization (Figure 3). The decrease in the consistency of the mixture due to the mixing step is consistent with the literature. In particular, it has been observed that as mixing times increase, samples of soy flour showed decreased complex viscosity dynamic moduli [19]. The homogenization process is often used on plant-based meat analog batter to create a more stable, uniform, and palatable product. This mechanical treatment, which often involves high pressure and intense shear forces, breaks down and evenly distributes the components of the paste. Homogenization significantly reduces the size of solid particles and liquid droplets (such as oil or fat) within the paste, creating a homogeneous and stable dispersion, and has a smoothing effect on the mixture. The mechanical and thermal stress can also cause structural changes in proteins and other macromolecules (like carbohydrates/fibers). This can lead to an increased water-binding capacity. The modification of proteins and the release of fibers enhance the batter’s capacity to bind water and stabilize the fat/water mixture, which is critical for forming a coherent patty structure. Evidence of this improved structure is demonstrated in Figure 3: although a decrease in consistency was noted, the phase angle of the Kenwood-treated sample was significantly lower than that of the untreated (“as is”) sample. This reduction in phase angle indicates a more structured and stable system in the homogenized mixture. Finally, by breaking down the plant cellular structure, the process can potentially increase the bioavailability of certain nutrients (like carotenoids or other bioactive compounds), making them easier for the body to absorb [20].

**Figure 4:**
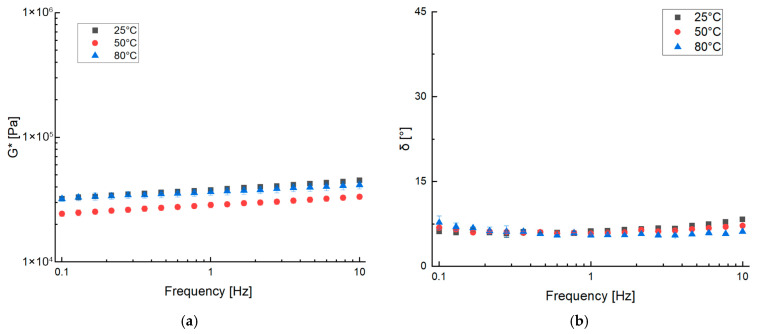
Frequency sweep test of ZBR at 25 °C, 50 °C and 80 °C measuring complex dynamic modulus $G^{*}$ (**a**) and phase angle δ (**b**).

Figure 4 shows the results obtained from frequency sweep tests at 25 °C, 50 °C, and 80 °C for the ZBR sample. When compared to the Ham sample data (Figure 1), the veggie burger paste exhibited lower consistency, which is reflected in the lower $G^{*}$ values. Despite this lower consistency, the mixture demonstrated good workability. Crucially, the phase angle results show a solid-like behavior, with values below 45°. These delta values are significantly lower than those reported for typical hamburgers, indicating the formation of a more rigid and highly structured matrix. This enhanced structuring is successfully achieved through the selection of thickening and structuring ingredients, with the final matrix organization attributed to the combined influence of proteins, starch, and fibers. Proteins are well-known for their structuring properties in various types of doughs and food matrices [21,22,23], but the presence of thickening agents such as starch and fiber, which create a stable and well-structured sample capable of resisting stress and frequencies [24,25], is fundamental, as reported in the literature [1].

**Figure 5:**
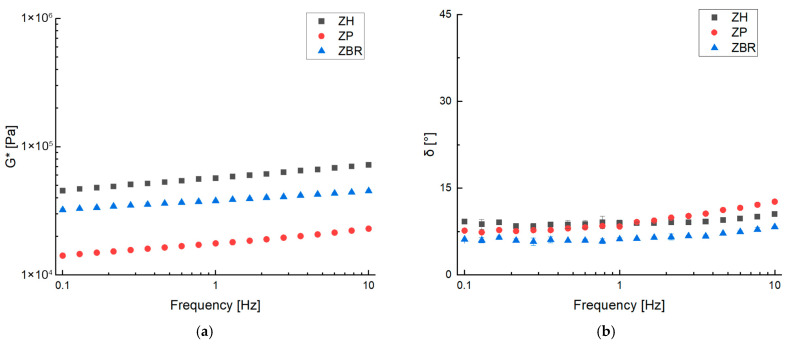
Frequency sweep test of ZH, ZP and ZBR at 25 °C measuring complex dynamic modulus $G^{*}$ (**a**) and phase angle δ (**b**).

The ZBR dough was prepared by replacing black rice proteins with hemp and pea proteins to see if they change the dough structure. The results ($G^{*}$ and phase angle) are shown in Figure 5. Despite the same protein concentration, the three mixtures had different consistencies. Compared to the ZBR sample, the mixture prepared with hemp proteins (ZH) had a higher consistency, even if it showed the same structuring degree (see phase angle values). The increased consistency observed may be attributable not to the protein type itself, but rather to the fiber content within the hemp flour, which significantly contributes to increasing $G^{*}$. The hemp protein isolate used in this formulation has a protein purity of only 50% *w*/*w*. The remaining composition includes 24% *w*/*w* fiber, 14% *w*/*w* fat, and 9.4% *w*/*w* carbohydrates. Consequently, achieving the target protein content necessitates incorporating additional hemp fiber, which is naturally present within the isolate. The literature supports the thickening and structuring effects of this fiber. For instance, studies on the addition of inulin demonstrate its important and desirable texturizing effect in achieving the right consistency for the final product [26].

A slight increase in the phase angle relative to the ZBR sample suggests the formation of a less structured dough. Furthermore, a comparison with the dough where black rice proteins were substituted with pea proteins revealed a distinct decrease in consistency. This decrease was quantitatively confirmed by lower $G^{*}$ values and a material exhibiting a greater dependence on frequency. The phase angle values are similar to those found for the ZH sample, suggesting that this sample was also less structured. Plant proteins mostly consist of glutenin, globulin, albumin, and prolamin, whose proportions differ in different proteins [27]. The ZBR sample showed the lowest frequency dependence, indicating a more structured compound, as confirmed by the lower phase angle values; this result confirms the better solubility of rice proteins at the mixing temperature inside the matrix during the sample preparation phase. At 80 °C, the solubility of plant proteins depends on many factors, such as pH and the solutes present. Furthermore, it also depends on the specific type of protein, since for some plant proteins, such as pea and hemp, the temperature can be high enough to induce thermal denaturation of the plant proteins and therefore partial denaturation. This latter phenomenon leads to a reduction in solubility and to aggregation or coagulation, and therefore to drastic solubilization [28,29].

**Figure 6:**
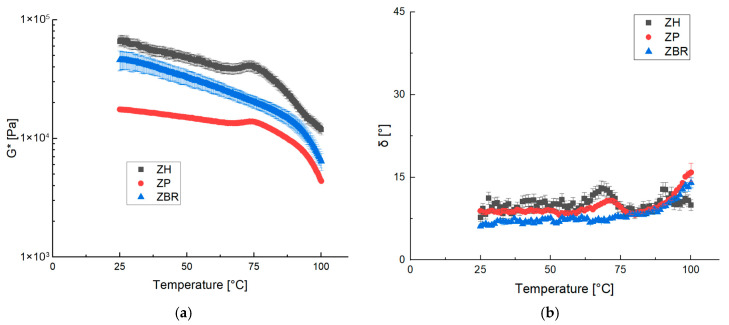
Complex dynamic modulus $G^{*}$ (**a**) and phase angle δ (**b**) of ZH, ZP and ZBR during curing.

The mechanical properties of the different preparations were evaluated using temperature ramp tests. All samples exhibited a complex modulus that steadily decayed across the entire temperature range analyzed, likely due to kinetic effects. Starch gelatinization was not observed in any of the studied samples. This absence is attributed to the low starch content in the dough, combined with the preparation protocol: the dough was prepared at 80 °C, a temperature sufficient to promote and complete gelatinization before the start of the rheological test. Instead, the observed peak is more likely related to protein denaturation, which occurs at approximately 70 °C for hemp and pea proteins, whereas higher temperatures are required for black rice proteins. For the ZH and ZP samples, a peak in the complex modulus and a simultaneous maximum in the phase angle were observed, which were linked to the denaturation of the proteins in the dough. The observed values of approximately 70 °C for the ZH sample and approximately 73 °C for the ZP are consistent with the literature [30]. However, for the black rice sample, the protein transition was not observed, which, according to the literature, occurs between 87 and 97 °C [31]. The thermal ramp probably does not allow for the visualization of the structural changes that occur at high temperatures; these are more evident from the frequency sweeps (Figure 6a), from which it was possible to observe that the value of the complex modulus increased after 50 °C and the slope decreased, indicating a structuring of the system.

The proteins utilized exhibited poor solubility within the inherent pH range of the sample matrix (6.30 to 6.85). However, the results strongly suggest that solubility significantly improved with increasing temperature. The literature supports this observation, with a reported denaturation temperature of approximately 80 °C for pea proteins and between 85.2 °C and 85.9 °C for hemp proteins, depending on the specific protein type and isolate [32,33]. The findings from the rheological tests indicate that the processing environment was suitable for protein solubilization and denaturation. This is notable because denaturation typically requires higher temperatures in less hydrated, lower-moisture environments. Therefore, raising the dough temperature is expected to promote the solubilization of proteins, a conclusion supported by the experimental results.

Heating the samples also imparted direct, beneficial effects on the proteins, as demonstrated by the temperature ramp test (Figure 6), particularly pea and hemp proteins. Their denaturation due to the thermal treatment has been shown to positively influence digestibility and enhance their ability to form desirable meat-like structures [31].

Dough systems often undergo extrusion processes, which promote the cleavage of hydrogen bonds and intramolecular disulfide bonds. Disulfide bonds are particularly important as they are primarily responsible for the rigidity of protein molecules and thus directly influence their functional properties [34]. This thermal and shear-induced breakdown leads to the formation of new protein aggregates, which ultimately provide the necessary final structure and texture to the product.

### 2.3. Rheological Data Interpretation

Rheological results of the frequency sweep test were interpreted using the weak-gel model in accordance with Equation (3). It is possible to see from Table 1 that the reference burger (Ham) always showed a higher consistency value (A parameter) than all the vegetable burgers. On the contrary, the structure parameter was always higher for the vegetable samples because of the different gelation and denaturation mechanisms occurring in them.

The cross-linking of the material is an important parameter, particularly for the stability of the compound and its processability, and it is related to the *z* parameter, which tends to increase with temperature due to improvements in cross-linking as the temperature rises. On the contrary, the increase in temperature has a weakening effect on parameter *A*, particularly at 50 °C, the temperature at which dough softening occurs due to kinetic effects.

By evaluating the ZBR sample, with and without the homogenization step, it was possible to observe that homogenization promoted the structuring of the sample (higher z) by lowering the system consistency (lower A) and it also improved its coherence and workability. Moreover, all the samples showed similar z values at 25 and 50 °C and showed increased of about 50% at 80 °C because of the increased structuring of the system with increased temperature, as observed in the time cure tests for samples ZP and ZH (Figure 6).

In particular, the lowest A value was observed for the dough with pea protein (ZP) at all temperatures, followed by the ZBR sample. The strength decayed with temperature, while the *z* parameter increased. The z values found for the vegetable burgers were always higher than those of the classic hamburger.

### 2.4. Cooking Tests and Appearance

**Figure 7:**
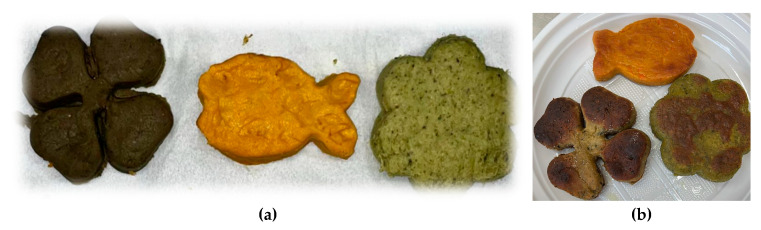
Shaped veggie burgers. From left to right: hemp protein-, pea protein-, and brown rice protein-based burgers before (**a**) and after (**b**) the cooking test.

The visible (external) appearance of the cooked and uncooked vegetable burgers is shown in Figure 7.

The external appearance of the reference meat burger and the vegetable burgers was homogeneous. However, the internal texture of the vegetable burgers was found to be finer than that of the meat burgers, both in the uncooked and cooked states. The meat burgers exhibited porous and rough internal structures both before and after cooking, which aligns with literature, which attributes this characteristic to the absence of specific texturing agents in the meat mixture [1].

Upon cooking, a browning effect caused by Maillard reactions involving proteins at high temperatures was observed, and a light crust formed on the outer layer. Compared to similar products described in the literature, our veggie burgers demonstrated a homogeneous, cohesive, and smooth surface, characteristics attributed to the blending processes. Furthermore, the samples successfully maintained their shape and water-retention capacity during storage, exhibiting gradual water release without noticeable cracking or breakage during the final cooking stage.

**Figure 8:**
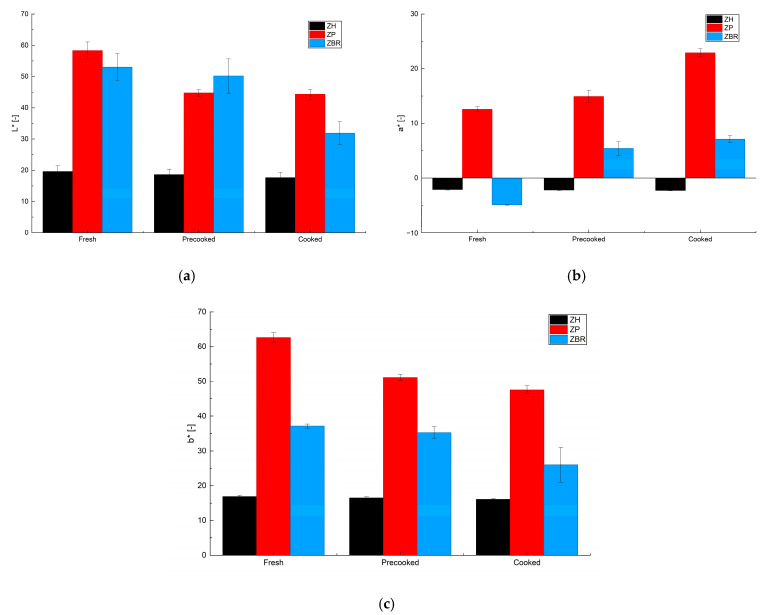
Color measurements in terms of L* (**a**), a* (**b**) and b* (**c**) parameters for three samples.

Color analysis was performed on the prepared samples—specifically in the fresh, precooked, and cooked states—by evaluating the L*, a*, and b* parameters, as defined in Section 4.4; the results are presented in Figure 8. Regarding the L* parameter (lightness), a decrease was observed during the cooking process. As anticipated, increasing the temperature during cooking promoted a browning effect typical of Maillard reactions. While this reaction also occurs in vegetable protein-based products, its intensity is generally less pronounced than in their meat-based counterparts [35]. This effect was significantly mitigated in the hemp protein-based sample (ZH), while the L* value remained nearly constant. The naturally dark color of the hemp proteins resulted in a darker initial mixture that underwent minimal change during thermal processing. Conversely, the a* values (redness/greenness) generally tended to increase during cooking, causing the samples to shift from a greenish hue toward a more reddish one. This effect, similar to the changes in L*, was attenuated in the ZH sample, which was less susceptible to these variations due to its intensely dark initial color. Finally, the b* parameter (yellowness/blueness) showed a significant decrease during cooking, resulting in a transition from an intense yellow color to a less pronounced one. There was also only a slight variation in the b* parameter in the ZH sample.

The color of vegetable burgers inherently differs from that of typical meat-based hamburgers, as confirmed by both visual inspection and documented L*, a*, and b* values reported in the literature [36]. For this reason, many formulations incorporate colorants to modify the hue and increase their similarity to meat products [37]. In the current work, however, we deliberately chose to retain the natural coloring of the plant-based ingredients, thereby promoting the authentic visual characteristics of plant-based products. The weight loss of the sample due to pre-cooking and cooking was also evaluated. The results indicate that all samples lost 20 ± 2% *w*/*w* of their initial weight during the pre-cooking phase in the oven and a further 10.5 ± 0.4% *w*/*w* during the final cooking phase. In total, compared to the fresh product, the weight loss was 29 ± 2% *w*/*w*.

## 3. Conclusions

This study successfully demonstrated that certain vegetable industry wastes, which remain rich in nutrients, can be effectively transformed into valuable by-products for the design of novel plant-based burgers. The strategic addition of proteins to a burger formulation provided added value to the preparation, enhancing both its nutritional profile and textural quality.

The use of three different protein types resulted in vegetable burger samples with varying mechanical properties. Specifically, the tests indicated that the burger formulated with black rice yielded a dough that was less consistent but exhibited a more highly structured matrix. This structural difference is likely attributable to the specific preparation temperatures and the holding times applied during the thermal processing.

Crucially, the final plant-based burgers demonstrated a pleasing external appearance and a sound final matrix, which was homogeneous in color and free of cracking caused by thermal stress. In conclusion, the samples produced using vegetable by-products are suitable for industrial processing and represent a compelling and sustainable alternative to conventional meat hamburgers.

## 4. Materials and Methods

### 4.1. Materials

Vegetable trimmings supplied by Gias S.p.a. (Mongrassano, Italy), such as zucchini, carrots, pumpkin, and broccoli, were used in this work. In addition to the vegetables, the following ingredients were used: commercial salt (Italkali, Milena, Italy); vegetable fiber, which was kindly provided by JRS Silvateam Ingredients srl (Rende, Italy); pea, hemp, and black rice proteins (Bulk, UK); potato starch, which was kindly provided by Gias S.p.a. (Mongrassano, Italy); and commercial sunflower oil (Fabiano, Rende, Italy). Ground beef and pork were purchased from a local market.

### 4.2. Veggie Burgers Preparation

The veggie samples were compared with a classic hamburger sample (Ham), which was prepared in the laboratory using minced veal and pork. The recipe was taken from the commercial hamburger label, and based on this, water was added (equal to 10% *w*/*w* of the meat and fiber). The fiber was solubilized in an amount equal to 2.5% *w*/*w* of the water at 50 °C and mixed using a magnetic stirrer. The ground meat was initially mixed on its own for 20 s. Subsequently, the water and fiber solution was added, and the mixture was mixed for 2 min using a Kenwood mixer (Kenwood Cooking Chef XL KCL95.424SI, Kenwood Limited, New Lane, Havant, UK). The final result was a heterogeneous mixture, which was denoted as Ham. A Zwick/Roell machine (Zwick/Roell ProLine, Ulm, Germany) was used to form a standard hamburger shape for rheological analysis; to obtain a standard height of 2 mm, a travel velocity of 2 mm/s was applied.

The plant-based burger samples were prepared using vegetable trimmings from zucchini, carrots, pumpkin, and broccoli. The trimmed vegetables were blended for 1 min using a commercial blender (Moulinette, Moulinex, France) to obtain a smooth purée with a moisture level of 90.80% ± 0.02%, which was determined using a thermobalance (Mettler Toledo, Greifensee, Switzerland). Then, the purée was heated to 80 °C using a heating plate (IKA C-MAG HS 7, IKA-Werke GmbH & CO. KG, Staufen, Germany) and fiber was added. The mixture was homogenized by a mechanical paddle stirrer (Heidolph RGL 100, Heidolph Instruments GmbH & Co. KG, Schwabach, Germany) and left to rest at 80 °C for 10 min. Starch was added under stirring, and subsequently, the correct quantity of proteins was introduced. Finally, all the other ingredients were added and the mixture was left to rest at 80 °C for 10 min before being homogenized with a Kenwood mixer (Kenwood Cooking Chef XL KCL95.424SI, Kenwood Limited, New Lane, Havant, UK) for 2 min.

Different formulations were then prepared, as shown in Table 2.

In all samples, a quantity of salt equal to 0.3% *w*/*w*, calculated based on the total weight, was added. Pea and black rice flours contain the same amount of protein (80%), while hemp flour contains 50%. Therefore, the hemp protein-based formulation (ZH) was formulated to have the same protein content as the other formulations while having a higher powder content than the other two recipes.

All the veggie burgers were made using specially printed molds and cooked.

Specifically, different shapes were created using molds and the different samples were precooked for a time equal to 25% of the total cooking time. After pre-cooking, they were immediately frozen at −18 °C for 15 days, after which, they were baked in an oven (Unox XF013, Stefania, Padova, Italy) under static conditions for 30 min at 180 °C.

### 4.3. Rheological Characterization

Rheological measurements were carried out using a rotational rheometer (Anton Paar MCR 702e, Anton Paar AG, Graz, Austria) with a parallel plate geometry (diameter = 25 mm; gap = 1.8 mm ± 0.1 mm) and a Peltier system for controlling the temperature. Preliminary stress sweep and time sweep tests were performed in the linear region. Subsequently, frequency sweep tests were performed at 25 °C, 50 °C and 80 °C in a 0.1–10 Hz frequency range, in the linearity. Furthermore, temperature ramp tests were performed from 20 °C to 100 °C, using a heating rate of 2 °C/min at 1 Hz in the linear region. Silicone oil (20 cSt; VWR Chemicals, Briare, France) was used to seal the sample edge in order to avoid water evaporation phenomena during tests. All measurements were conducted twice.

The results are reported as complex dynamic modulus $G^{*}$ and phase angle δ, evaluated as

(1) $$G^{*}=\sqrt{\left({G^{\prime }}^{2}+{G^{\prime \prime }}^{2}\right)}$$

(2) $$\delta =\tan^{-1}\left(\frac{G^{\prime \prime }}{G^{\prime }}\right)\;$$

The frequency sweep tests were interpreted using the weak gel model:

(3) $$G^{*}\;=\;A\omega^{\frac{1}{z}}\;$$

where $G^{*}$ is the complex modulus, ω is the frequency, A is a measure of gel strength, and z is a measure of gel structuration [38].

### 4.4. Appearance Observation

Qualitative assessments of the veggie burgers were carried out using visual inspections and taste tests. Alongside these assessments, color measurements were conducted on the freshly prepared (fresh) samples, after pre-cooking in an oven (pre-cooked), and after final cooking in a pan (cooked). The measurements were taken three times using a digital colometer (Croma Meter CR-400, Konica Minolta, Tokyo, Japan). Preliminary calibration was performed using a standard white support. All measurements were analyzed in the CieLab space, where L* is the axis of lightness; a* is the axis of the red–green transition; and b* is the axis of the yellow–blue transition. The weight loss (%wl) of each sample was also evaluated by weighing it on an analytical balance at the fresh (w_i_), pre-cooked, and post-cooked stages (identified as w_i_) [39]. The results are reported in percentages according to Equation (4):

(4) $$\%wl\;=\left(\frac{{w_{f-}w}_{i}}{w_{i}}\right)*100\;$$

### 4.5. Data Treatments

All samples were produced in triplicate, and three runs of each test were performed on each sample. Additionally, the data were statistically analyzed. All the rheological data were processed and fitted with the model equations in Section 4.3 using the software OriginPro (Version 2021b, OriginLab Corporation, Northampton, MA, USA). With the same software, the differences between data were evaluated using a one-way analysis of variance (ANOVA) test at a 5% significance level and a Tukey test for mean comparisons. In the results, the values at different temperatures and as the values of the different types of dough were compared. Therefore, for each value, there are two letters indicating the significance of the values; the first letter (lowercase) indicates the comparison between temperatures and the second letter (uppercase) indicates the comparison between the different types of dough.

## Figures and Tables

**Table 1:** Weak-gel parameters A (Pa*s^1/z^) and z (dimensionless). Different letters for the same parameter indicate significantly different values. Lowercase letters refer to variations between different temperatures while uppercase letters refer to variations between different types of dough.

ID	A, 25 °C	z, 25 °C	A, 50 °C	z, 50 °C	A, 80 °C	z, 80 °C
Ham	80,000 ± 3000 ^a,A^	7.14 ± 0.06 ^a,A^	78,000 ± 700 ^a,A^	7.38 ± 0.09 ^a,A^	142,000 ± 3000 ^b,A^	10.49 ± 0.09 ^b,A^
ZBR *	49,190 ± 1000 ^a,B^	10.00 ± 0.13 ^a,B^	36,800 ± 1000 ^b,B^	10.99 ± 0.09 ^b,B^	37,800 ± 300 ^b,B^	17.03 ± 0.06 ^c,B^
ZBR	38,100 ± 700 ^a,C^	13.96 ± 0.04 ^a,C^	29,000 ± 1000 ^b,C^	14.95 ± 0.08 ^a,C^	36,000 ± 1000 ^a,B^	18.59 ± 0.09 ^b,BC^
ZP	17,800 ± 400 ^a,D^	9.71 ± 0.08 ^a,B^	16,200 ± 700 ^a,D^	10.67 ± 0.01 ^a,C^	12,300 ± 100 ^b,C^	15.14 ± 0.65 ^b,C^
ZH	57,200 ± 1000 ^a,E^	10.09 ± 0.13 ^a,B^	46,000 ± 1000 ^b,E^	10.76 ± 0.11 ^a,C^	54,800 ± 900 ^a,D^	14.07 ± 0.73 ^b,C^

* sample without Kenwood step.

**Table 2:** Veggie burger formulations.

Ingredient %*w*/*w*	ZBR	ZP	ZH
Vegetable puree	67	67	67
Fiber	3	3	3
Water	0	0	0
Potato starch	15	15	15
Black rice protein	10	0	0
Pea protein	0	10	0
Hemp protein	0	0	16
Sunflower oil	5	5	5
Salt	0.3	0.3	0.3

## Data Availability

The data used to support the findings of this study can be made available by the corresponding author upon request.
